# An Efficient IAKF Approach for Indoor Positioning Drift Correction

**DOI:** 10.3390/s22155697

**Published:** 2022-07-29

**Authors:** Shang-Hsien Lin, Hung-Hsien Chang Chien, Wei-Wen Wang, Kuang-Hao Lin, Guan-Jin Li

**Affiliations:** 1 Systems Development Center, National Chung-Shan Institute of Science and Technology, Taoyuan 325, Taiwan; hunghsieh@ncsist.org.tw (H.-H.C.C.); jimi@ncsist.org.tw (W.-W.W.); 2 Department of Electrical Engineering, National Formosa University, Yunlin 632, Taiwan; khlin@nfu.edu.tw (K.-H.L.); 10765117@gm.nfu.edu.tw (G.-J.L.)

**Keywords:** indoor positioning system, ultra-wideband, Kalman filter, RSSI, AoA

## Abstract

In this study, an indoor positioning shift correction architecture was developed with an improved adaptive Kalman filter (IAKF) algorithm for the people interference condition. Indoor positioning systems (IPSs) use ultra-wideband (UWB) communication technology. Triangulation positioning algorithms are generally employed for determining the position of a target. However, environmental communication factors and different network topologies produce localization drift errors in IPSs. Therefore, the drift error of real-time positioning points under various environmental factors and the correction of the localization drift error are discussed. For localization drift error, four algorithms were simulated and analyzed: movement average (MA), least square (LS), Kalman filter (KF), and IAKF. Finally, the IAKF algorithm was implemented and verified on the UWB indoor positioning system. The measurement results showed that the drift errors improved by 60% and 74.15% in environments with and without surrounding crowds, respectively. Thus, the coordinates of real-time positioning points are closer to those of actual targets.

## 1. Introduction

Intelligent Following systems installed on carts have widespread use on farms and shopping malls [1]. In the present study, time of flight (ToF) was used to develop a distance estimated architecture. The advantage of ToF is that it is free from the time errors caused by differences in the internal calculations of the anchors and the tag. After the anchor–tag distance is determined, the Kalman filter algorithm can be used to converge sensor data and eliminate errors caused by noise. Obtaining anchor distance and angle to the tag enables the system to determine the tag’s relative position and control the cart to follow the tag. Because the follower system cannot provide the location in the vast field, the management center is disabled to control the real-time information of the vehicle location. Therefore, the current UWB technology was used to expand the positioning function. To allow the tag to be positioned clearly, this study proposed the development of an indoor positioning shift correction architecture based on the improved adaptive Kalman filter (IAKF) algorithm.

Outdoor and indoor positioning technologies have a wide range of applications. Drones, vans, and self-driving cars rely on positioning systems to perform functions such as satellite navigation, one-button four-rotor return flight, car parking location determination, and auto-navigation. Most common indoor positioning systems [2,3] use received signal strength indicator (RSSI) [4,5] as the distance calculation method. Although the development cost of distance calculation technology is relatively low, its wireless transmission frequency is 2.4 GHz, which is susceptible to Wi-Fi signal interference and causes positioning errors [6]. The angle of arrival (AoA) [7] distance calculation method involves the use of array antennas, and the absolute position coordinates are calculated according to the signal return angle; nevertheless, the overall cost is the highest among all indoor positioning systems, due to the high cost of array antennas. An ultra-wideband (UWB) indoor positioning system was employed in this study. The system uses the time difference of arrival (TDoA) positioning method [8,9,10] to calculate the distance between the anchor and the tag. The signal transmission frequency range of the system is 3.1–10.6 GHz [11,12,13], which is less susceptible to interference by 2.4-GHz Wi-Fi signals. The TDoA [14,15] positioning method does not influence the positioning accuracy because of signal strength weakening. UWB indoor positioning systems are employed in various applications [16,17,18,19,20], such as unmanned smart object–finding systems in supermarkets, navigation of airport terminal lobbies and complex station maps, and security positioning for elderly care centers, due to their high transmission rate and low power consumption. Elsanhoury [21] presented a compact review focusing on using multi-sensor fusion-based systems in specific applications. The researcher designed a concise tutorial for researchers seeking an overall view of UWB positioning technology. Schwarzbach [22] discussed a procedure which allows the synthetic generation of UWB distance measurements with respect to theoretical error sources and parameterization based on a conducted measurement campaign.

In this study, the problem of shifting of the positioning point observed in UWB indoor positioning systems in indoor spaces was analyzed. A UWB indoor positioning system was used in the experimental method to locate the target point. MATLAB was used to analyze the shifting error of the positioning point. In addition to external environmental noise, UWB indoor positioning systems also generate signal processing noise. The noise causes shifting of the positioning point, decreasing the positioning accuracy. Therefore, four algorithms were employed in this study to address the problem of shifting of the positioning point: moving average, least square, Kalman filter, and IAKF. Then, the actual measured coordinate points were input into the algorithms to decrease the shifting of the positioning point.

For the experimental analysis, the IAKF algorithm was employed. The algorithm was installed in the firmware of the UWB indoor positioning system (IPS). Based on the test conditions, the experimental analysis was mainly divided into static positioning analysis (analyzing the signal processing noise generated by the UWB IPS), human interference factor positioning analysis (analyzing the external environmental factors), and dynamic positioning analysis (analyzing the real-time positioning performance and noise processing capabilities). The experimental conditions were in line with the actual scenario encountered by the IPS during positioning. The IAKF algorithm was found to improve the shift error of the positioning point, thereby serving the purpose of decrease in shift error distance and increase in positioning accuracy.

UWB IPSs comprise four anchors: a coordinator, a router, a server, and a tag. In the positioning process, these anchors are used to measure the distance to the static tag. The anchors return the ranging values to the coordinator and then return the ranging data to the server through the router for triangulation calculation [23,24,25]. Finally, the position coordinates of the positioning target are obtained.

The remainder of this paper is organized as follows. Section 2 describes the system architecture design. In Section 3, the algorithms employed to address the positioning point shift problem are described, and the simulation results are presented and compared. In Section 4, the experimental results are presented, and positioning performances are compared and discussed. Finally, in Section 5, the conclusions are presented.

## 2. System Architecture

**Figure 1:**
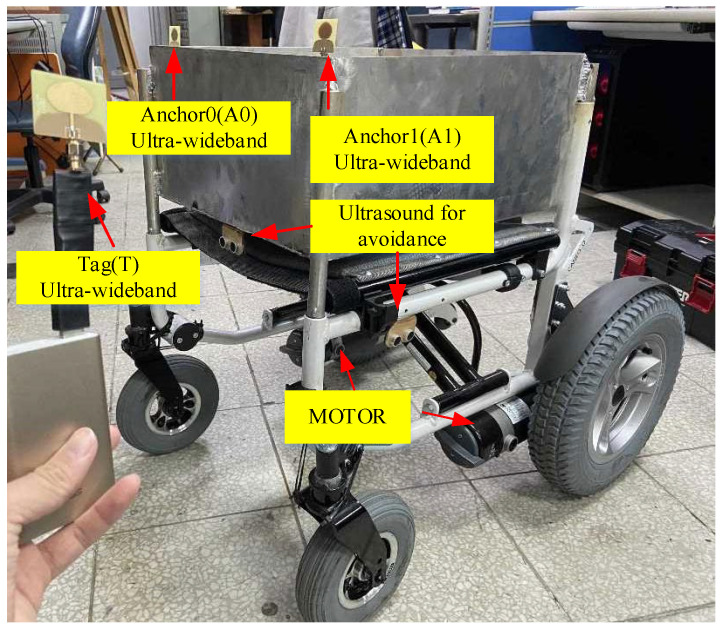
Block diagram of intelligent following system hardware.

**Figure 2:**
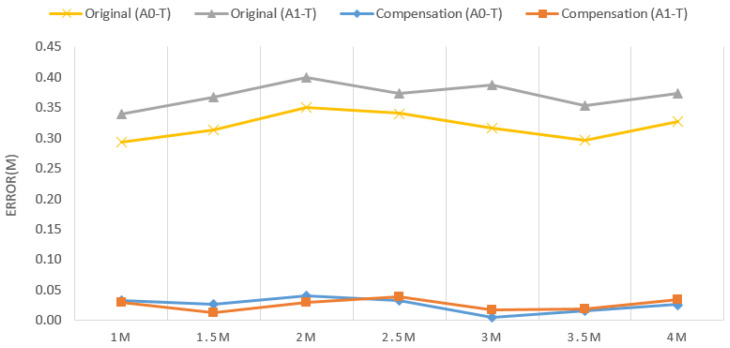
Distance errors before and after compensation.

Figure 1 presents a typical following system comprising three UWB sensors, one on each lateral side of the cart (Anchor0 & Anchor1) and the third carried by the user (Tag). The demand for high precision and long endurance makes UWB the first choice for wireless sensors. To prevent collisions caused by sensing errors, ToF and Kalman filter algorithms were employed for error convergence. Furthermore, an error compensation algorithm that substantially reduces the magnitude of errors was also proposed. Finally, distance and angle formulae were applied to determine the user’s position relative to the cart. Actual measurement results indicate that, after optimization by the sensing error compensation algorithm, the error in cart–tag distance was reduced from 29–40 cm to within 5 cm for any distance and angle (Figure 2), thus confirming the effectiveness of the algorithm.

**Figure 3:**
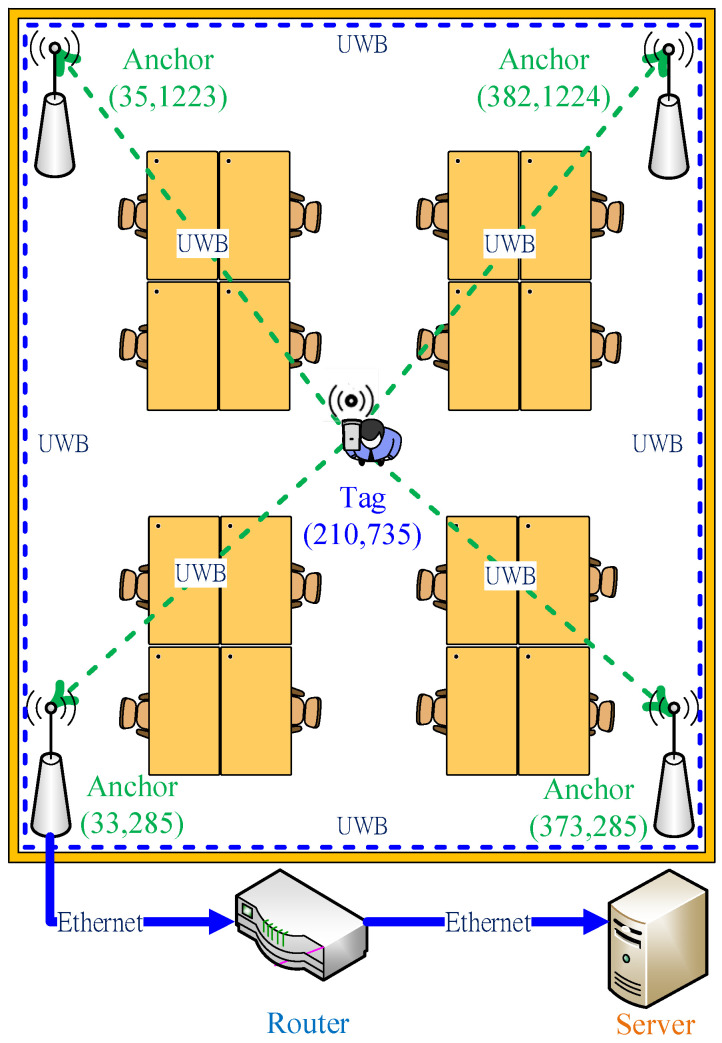
The system architecture of ultra-wideband (UWB) indoor positioning systems.

Although the following system can accurately obtain the information of distance and angle, it cannot provide the position of the user (Tag). Therefore, anchors were set around the environment according to the circuit presented in Figure 1, and relevant distance information between the tag and anchors was obtained for the positioning computing. The system architecture of the UWB indoor positioning system is shown in Figure 3. This system architecture mainly consists of four anchors, a coordinator, a router, a server, and a tag. The hardware positioning process of the indoor positioning system was as described herein. Four anchors were used to measure the distance from the tag. All anchors send the distance value to the coordinator, which then sends the received distance information to the server through the router for the triangulation positioning algorithm to obtain the absolute position of the tag. Finally, the hardware architecture process of the positioning function of the UWB indoor positioning system was completed.

## 3. Improvement in Algorithm of Positioning Point Shift and Simulation Results

In this section, the moving average method, least square method, Kalman filter algorithm, and proposed IAKF algorithm are discussed.

For the source of material analyzed by the algorithm, the anchor and tag in the system architecture shown in Figure 3 are used to calculate the distance information through ToF. Then the information is sent to the server through the Ethernet for a triangulation positioning algorithm to calculate the position of the original measurement (*x*-axis and *y*-axis coordinates). UWB IPS causes the floating phenomenon of positioning points and reduces the positioning accuracy because the wireless communication system is affected by noise and the environment. Then, four algorithms are used to simulate and analyze the positioning coordinates of the original measurement and compare the positioning improvement range of each algorithm. Finally, an algorithm that can best improve the positioning is determined for use in this UWB IPS positioning system. The algorithm is used mainly to perform a software algorithm by C language program in the server so that the tag can obtain more accurate coordinate positions during actual measurement.

### 3.1. The Moving Average Method

In the moving average method [26], a set of recently sampled data are used to predict the trend of future data. This algorithm is most commonly used in data smoothing methods. One or a certain number of values are calculated based on a time series to predict the trend of data. Therefore, when the value of a time series is affected by random fluctuations, it becomes difficult to understand the trend of a series of data. The moving average method eliminates random fluctuations in the data so that the future data trend can be predicted accurately. The non-weighted average of *N* samples before the current input (including the current input) is considered in this method as follows:

(1) $$y\left(n\right)=\frac{1}{N}\overset{N-1}{\underset{i=0}{\sum }}x\left(n-i\right)$$

### 3.2. The Least Square Method

The least-square method [27] is an algorithm for data smoothing. It finds the best matching function by minimizing the sum of squared errors. The least-square method can quickly predict the future trend of the data:

(2) $$\mathrm{LS}=\frac{1}{N}\overset{N}{\underset{i=1}{\sum }}{\left(y_{i}^{p}-y_{i}\right)}^{2}$$

where *N* is the number of sampled data points, $y_{i}^{p}$ is the actual measured value, and $y_{i}$ is the target value. The position of the tag is $\left(x_{m},y_{m}\right)$. The position coordinates of the anchor are $\left(x_{1},y_{1}\right),\left(x_{2},y_{2}\right),\left(x_{3},y_{3}\right)$. However, triangulation and least square methods are used for calculation. The equations used for the least square method are as follows:

(3) $$\{\begin{array}{c} {\left(x_{m}-x_{1}\right)}^{2}+{\left(y_{m}-y_{1}\right)}^{2}=d_{1}^{2}\\ {\left(x_{m}-x_{2}\right)}^{2}+{\left(y_{m}-y_{2}\right)}^{2}=d_{2}^{2}\\ {\left(x_{m}-x_{3}\right)}^{2}+{\left(y_{m}-y_{3}\right)}^{2}=d_{3}^{2} \end{array}$$

### 3.3. Kalman Filter Algorithm

**Figure 4:**
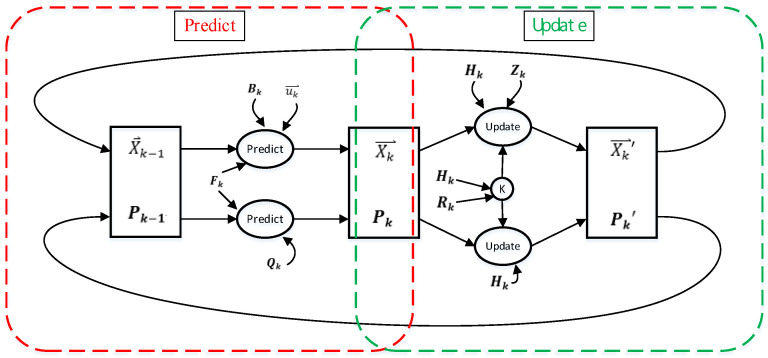
Kalman filter algorithm calculation flow chart.

The Kalman filter [28] algorithm was proposed in the paper titled “A New Approach to Linear Filtering and Prediction Problems”, published by Hungarian émigré Rudolf E. Kálmán in 1960. The algorithm uses phase-locked loops, a technology that uses feedback control to achieve frequency and phase synchronization. The algorithm is used in many communication devices, such as frequency-modulation radios. The basic dynamic system of the Kalman filter algorithm can be represented using a Markov chain model, where linear transformation is interfered with by Gaussian noise. The system state can be represented by a vector whose element is a real number, which is added to each time interval in discrete time. This linear transformation operates on the current state and then produces a new state. During state transformation, some noise is produced. System control factors are also added to the system. Another linear transformation interfered by noise produces the output states. Kalman filter algorithm is an optimized discrete data recursive algorithm, and the flowchart is shown in Figure 4. The estimated value of the previous state and the measured value of the current state are convoluted to calculate the estimated value of the current state. After each recursion, the estimated value is closer to the dynamic system state, resulting in a smoother data trend [29].

The equation of the Kalman filter algorithm can be written as follows:

(4) $$\vec{X\;}=F\cdot \vec{X_{d}}+B\cdot \vec{u}$$

where $\vec{X\;}$ is the predicted value vector, F is the optimized prediction matrix, $\vec{X_{d}}$ is the previous state estimate vector, B is the control factor matrix, and $\vec{u}$ is the external environmental factor vector. Furthermore,

(5) $$P=F\cdot P_{d}\cdot F^{T}+Q$$

where P is the covariance matrix between the predicted value and the last state estimate, $P_{d}$ is the covariance matrix between the last estimate and the last actual measured value, and Q is the noise matrix of the external environment uncertainty.

(6) $$K=P\cdot H^{T}\cdot {\left(H\cdot P\cdot H^{T}+R\right)}^{-1}$$

where K is the Kalman gain, H is the measurement parameter matrix, and R is the sensing noise matrix generated by the sensor.

(7) $${\vec{X^{\prime }}}^{\;}=\vec{X\;}+K\cdot \left(\vec{Z\;}-H\cdot \vec{X\;}\right)$$

where $\vec{X^{\prime }}$ is the new estimated value after an arithmetic operation and $\vec{Z\;}$ is the actual measurement value matrix.

(8) $$P^{\prime }=P-K\cdot H\cdot P$$

where $P^{\prime }$ is the covariance matrix of the new estimated value and the actual measured value.

### 3.4. Improved Adaptive Kalman Filter Algorithm

**Figure 5:**
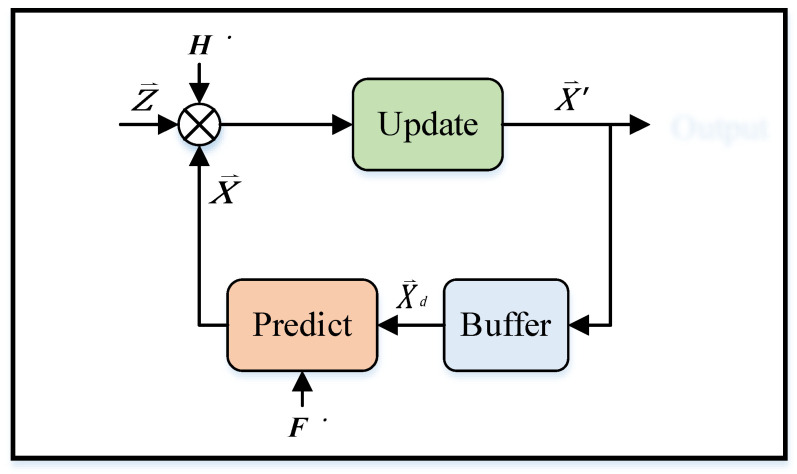
The architecture of the improved adaptive Kalman filter (IAKF) algorithm.

The Kalman filter can only predict the best estimate of the target point under the linear Gaussian model. However, in practice, UWB IPSs always have some nonlinear interference noise. The noise of the nonlinear function may exhibit a square relationship, logarithmic relationship, exponential relationship, trigonometric function relationship, etc. Some nonlinear functions can be converted into approximate linear functions by using linear differential equations. However, the system state cannot be estimated in certain nonlinear systems, such as missile trajectory estimation systems, aircraft flight paths, satellite positioning navigation, and indoor positioning system. In nonlinear systems, the nonlinear shift factor of the positioning point cannot be ignored; the system cannot achieve real-time calculation, which causes positioning delay and positioning point shift. Therefore, in this study, IAKF, an improved algorithm, was proposed to tackle the positioning point shift problem in nonlinear systems. The IAKF algorithm uses the linearization method and converts a nonlinear system into an approximate linear filtering problem based on the Kalman filter. The optimized prediction matrix F and the measurement parameter matrix H of the Kalman filter are expanded into the Taylor series. Next, to establish an approximate linearized mathematical model, the Jacobi matrix is calculated to omit terms above the second order. Thus, the IAKF algorithm solves the limitation of the Kalman filter, which cannot be applied to nonlinear systems. The IAKF architecture is shown in Figure 5. Assume that the state variable has *n* dimensions, $x\left(k\right)={\left[x_{1}\;x_{2}\;\ldots \;x_{n}\right]}^{T}$; then the Jacobi matrix [30] can be calculated as follows:

(9) $$F^{\prime }=\frac{\partial F}{\partial x}=\left[\begin{array}{ccc} \frac{\partial f_{11}}{\partial x_{1}}\; & \cdots & \frac{\partial f_{1n}}{\partial x_{n}}\\ \vdots & \ddots & \vdots \\ \frac{\partial f_{n1}}{\partial x_{1}} & \cdots & \frac{\partial f_{nn}}{\partial x_{n}} \end{array}\right]$$

(10) $$H^{\prime }=\frac{\partial H}{\partial x}=\left[\begin{array}{ccc} \frac{\partial h_{11}}{\partial x_{1}}\; & \cdots & \frac{\partial h_{1n}}{\partial x_{n}}\\ \vdots & \ddots & \vdots \\ \frac{\partial h_{n1}}{\partial x_{1}} & \cdots & \frac{\partial h_{nn}}{\partial x_{n}} \end{array}\right]$$

### 3.5. Simulation Results

MATLAB was used to realize the analyzed positioning point shifting error and improvement in the results. Four algorithms were used in the simulation process: moving average method, least square method, Kalman filter algorithm, and the IAKF algorithm. The shifting error of the positioning point was analyzed through simulation and the improvement range of the positioning point shift achieved by each algorithm was compared. The simulation environment of the UWB IPS involved four anchors for the measurement of the positioning point of a static tag. The coordinate values were measured every second by the IPS for a total of 30 s; thus, 30 coordinates were obtained. Next, the shifting of the positioning point was analyzed. For an improved simulation of positioning point shifting, the actual measured coordinate values were substituted into MATLAB, and the positioning point shifting of the x and y coordinates was analyzed. The simulation environment was the same as that shown in Figure 3.

**Figure 6:**
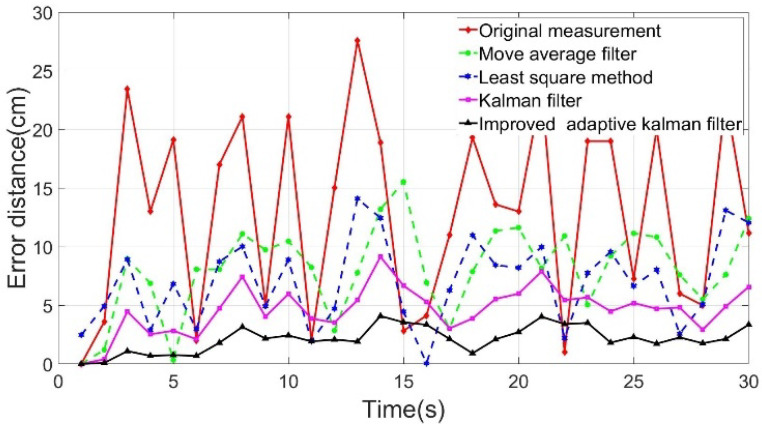
Shifting error distance analysis chart.

Figure 6 shows the positioning point shift error distance yielded by the algorithms. The horizontal axis is the time axis (unit: s), and the vertical axis is the shift error distance (unit: cm) of the positioning point. The deviation distances of the positioning point of all algorithms were compared and revealed that the IAKF algorithm yielded the line with the smoothest positioning point shift error distance.

The average shift error distance (ASED) obtained using the IAKF algorithm reduced from 12.91 to 2.13 cm, showing an improvement of 83.5% (Table 1). Therefore, compared with other algorithms, the best improvement was observed using the IAKF algorithm. The equation of improvement is defined as

(11) $$\mathrm{Improvement}=\frac{\mathrm{Original}\;\mathrm{ASED}\;-\;\mathrm{IAKF}\;\mathrm{ASED}}{\mathrm{Original}\;\mathrm{ASED}}\times 100\%$$

## 4. Experiment Results

In this section, the measurement results obtained using the IAKF algorithm on the UWB IPS are presented to demonstrate the reduction in positioning point shift. The test environment was set as static (with/without surrounding interference) and dynamic.

### 4.1. Improvement Result of Static Positioning Point Shifting

The test environment of the static positioning point shifting analysis was the same as that shown in Figure 3, which included four anchors to locate a static tag. The experimental method involved measuring the positioning point coordinates of the static tag in an indoor positioning test environment. The shifting of the tag was analyzed in the static state and in the absence of external interference factors.

**Figure 7:**
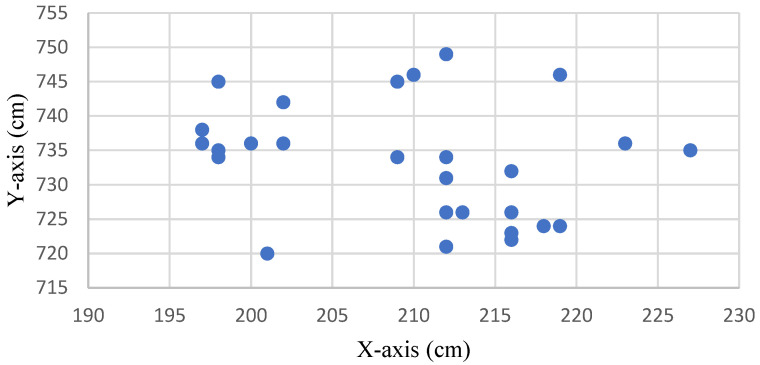
Distribution of the original measurement positioning points without drift correction.

**Figure 8:**
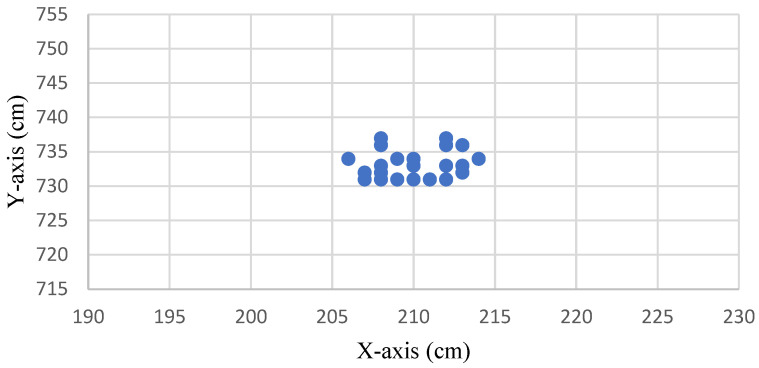
Distribution of the improved positioning points after being converged using the IAKF algorithm.

Figure 7 shows the distribution without drift correction of the original measurement positioning point at the coordinate (210, 735). The positioning point signal was converged using the IAKF algorithm, as shown in Figure 8.

**Figure 9:**
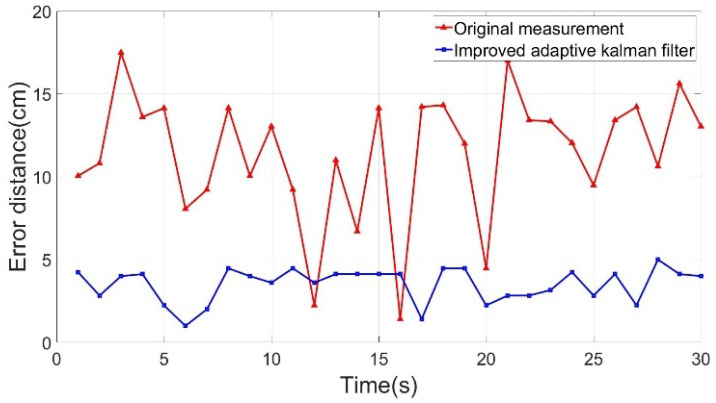
Shifting error distance analysis chart of the static positioning point.

The static assumption was used as a priori knowledge to obtain the results in Figure 9. The ASED of the positioning point before improvement was 11.42 cm, and 3.50 cm after correction. In Figure 9, the orange line indicates that the shift error distance after correction tends to be smooth, and the improvement rate is 69.33% compared with the original signal.

### 4.2. Result of Improved Surrounding Crowd’s Influence

**Figure 10:**
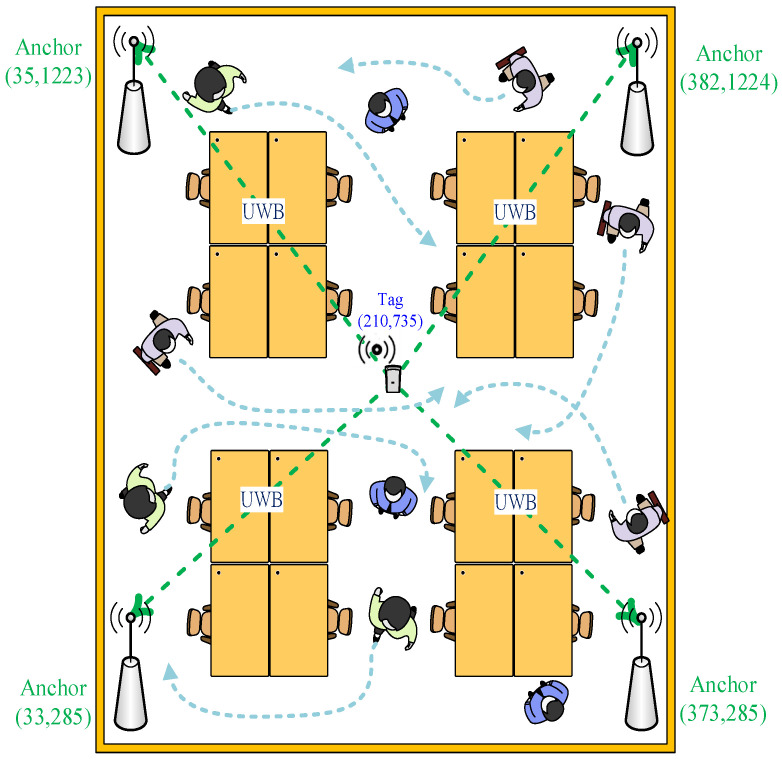
Experimental environment diagram of the people interference point shifting error analysis.

The test environment of positioning point shifting analysis for the people interference condition is shown in Figure 10. Here, the positioning point coordinates of a static tag in an indoor positioning test environment were measured. Approximately 1–10 people were asked to walk around the positioning space to create people interference factors. Then, the impact of positioning point shifting caused by people moving in the positioning space was analyzed. The actual measurement values and the shifting of the positioning point were analyzed. Finally, the improvements achieved after interference by different numbers of people were compared.

**Figure 11:**
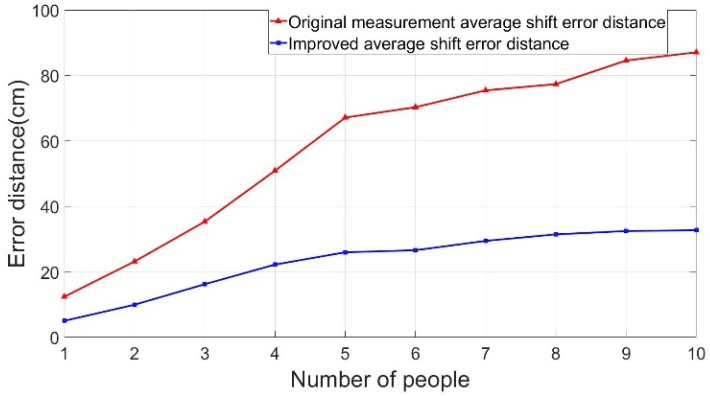
The average shift error distance (ASED) analysis diagram of people interference.

Figure 11 shows the ASED of the positioning point for various people interference factors (1–10 people). The blue line shows a considerably lower (by 50–60%) ASED compared with the original ASED denoted by the orange line.

### 4.3. Improvement Result of Dynamic Positioning Point Shifting

**Figure 12:**
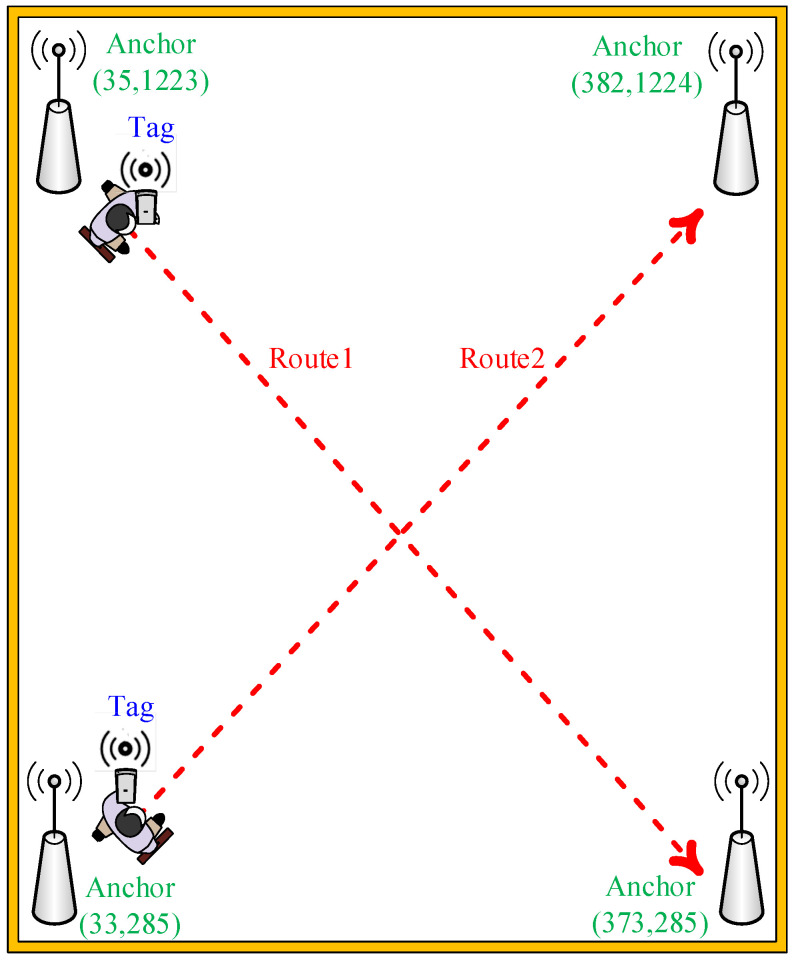
Experimental environment diagram of dynamic interference point shifting error analysis.

The test environment of dynamic interference positioning point shifting analysis is shown in Figure 12. Four anchors were used to locate the moving tag in an indoor positioning space to analyze the movement trajectory of the tag. The actual positioning point movement trajectory was set as routes 1 and 2. The results reveal that the IAKF algorithm yielded improved dynamic positioning point-shifting error.

**Figure 13:**
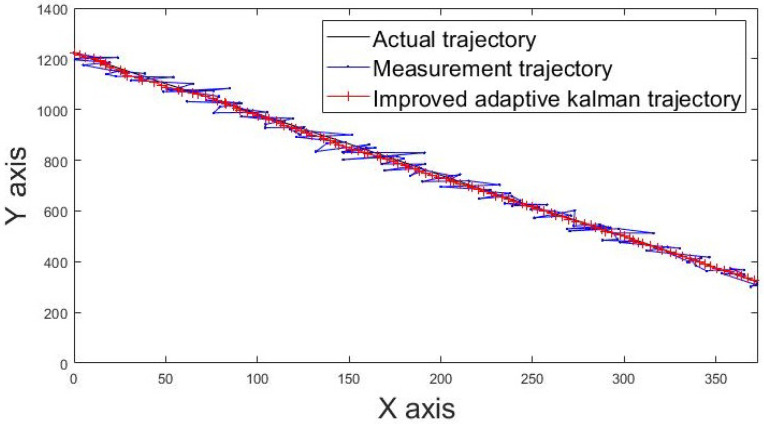
Movement trajectory 1 of tag.

**Figure 14:**
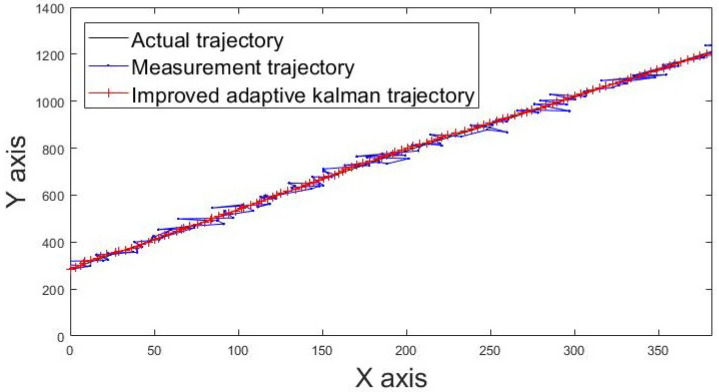
Movement trajectory 2 of tag.

The actual movement trajectories 1 and 2 of the static tag are shown in Figure 13 and Figure 14. The blue line indicates the movement trajectory of the original measurement. The red line shows the improved movement trajectory achieved using the IAKF algorithm; the red line is closer to the ideal trajectory shown by the black line, thus indicating that the IAKF algorithm successfully tackles the dynamic positioning point shifting problem.

**Figure 15:**
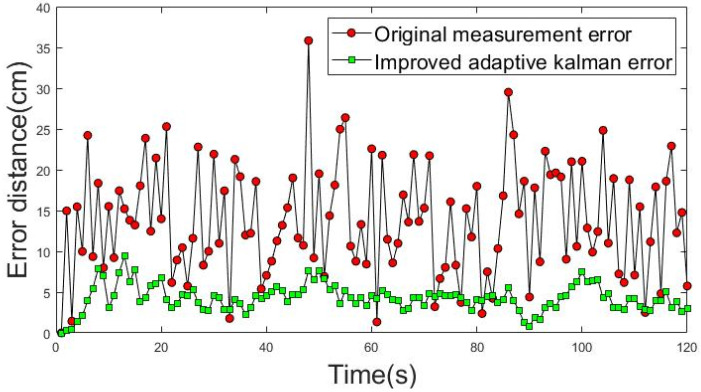
Shift error distance analysis of movement trajectory 1.

**Figure 16:**
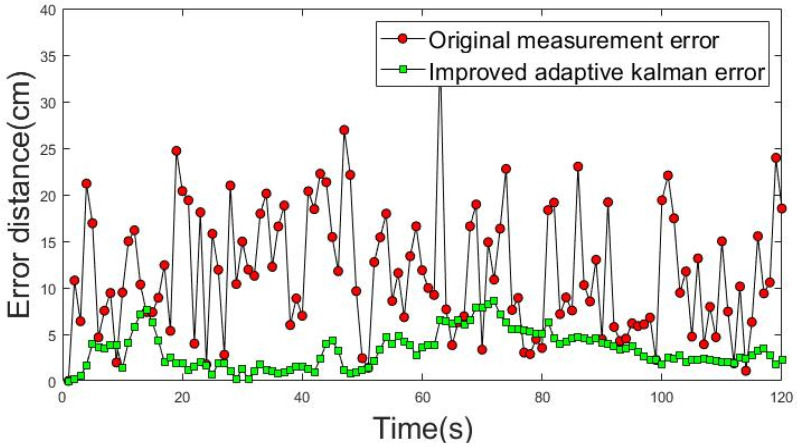
Shift error distance analysis of movement trajectory 2.

An ideal uniform rectilinear motion was used as a priori knowledge to obtain the results in Figure 15 and Figure 16. The movement trajectories 1 and 2 of dynamic positioning point shift error analysis are shown in Figure 15 and Figure 16. The red dotted line shows the actual measurement values of the shift error distance of the dynamic positioning point, and the green dotted line shows the improved shift error distance of the positioning point achieved using the IAKF algorithm.

The average shift error distance (ASED) of the positioning point before the improvement in movement trajectory 1 was 13.19 cm. The ASED of the improved positioning point was 3.87 cm and the percentage improvement was 70.63%. The ASED of the positioning point before the improvement of movement trajectory 2 was 12.86 cm. The ASED of the improved positioning point was 2.91 cm; thus, the percentage improvement was 77.36%.

Table 2 shows that the ASED obtained using the IAKF algorithm was 2.25 cm, with an improvement of 74.15%. Compared with the improvement results of other algorithms, the shift error distance obtained using the IAKF algorithm is the shortest.

## 5. Conclusions

In this study, there was an improvement in the positioning point shift. In addition, the IAKF algorithm was successfully implemented in the UWB indoor positioning system, and the shifting of positioning points caused by environmental and other factors in the UWB IPS was analyzed. As a result, there was a significant decrease in the shift error distance of the positioning point. Using the IAKF algorithm, the real-time positioning measurement point was closer to the actual target point, thereby showing improved positioning accuracy and system stability in the UWB IPS.

## Figures and Tables

**Table 1:** Comparison of average drift error distance and improvement range.

Algorithm	Average Shift Error Distance (ASED)	Improvement (%)
Original Measurement	12.91 cm	N/A
Moving Average	8.05 cm	37.65%
Least Square	7.01 cm	45.7%
Kalman Filter	4.65 cm	63.98%
Improved Adaptive Kalman Filter	2.13 cm	83.5%

**Table 2:** Comparison of improvement results obtained using different algorithms.

	This Work	[31]	[32]	[33]	[34]
Original ASED (cm)	8.69	30~34	21.35	74.46	88
Improved ASED (cm)	2.25	18~20	10.189	57.25	26
Improvement Percentage (%)	74.15	40.76	52.28	23.11	70.45

## Data Availability

The datasets generated during and/or analyzed during the current study are available from the corresponding author on reasonable request.
